# The Successful Management of Severe Leptospirosis by Using Meropenem and Corticosteroids in the Setting of Multi-Drug Hypersensitivity: A Case Report

**DOI:** 10.7759/cureus.92921

**Published:** 2025-09-22

**Authors:** Nikhil Deans, Merary Merybal, Jeric Ashwin, Roshan Ajoy Kadavan

**Affiliations:** 1 General Internal Medicine, Clinician Care Hospital, Chennai, IND; 2 Anaesthesiology, Clinician Care Hospital, Chennai, IND; 3 Otolaryngology, Clinician Care Hospital, Chennai, IND

**Keywords:** complications of leptospirosis, leptospirosis with severe clinical manifestation, meropenem therapy, severe leptospirosis, weil's disease, zoonotic infections

## Abstract

Leptospirosis is a globally significant zoonotic infection that poses a major burden on tropical regions. It is often misdiagnosed due to its highly variable clinical spectrum. Although severe leptospirosis is classically characterized by jaundice, acute kidney injury (AKI), and severe pulmonary hemorrhage syndrome (SPHS), atypical anicteric presentations pose diagnostic challenges and delay treatment initiation, increasing mortality risk. We report the case of a 23-year-old female with severe leptospirosis, which presented a significant therapeutic challenge due to hypersensitivity to all first-line antimicrobial agents for leptospirosis, limiting treatment options. Her clinical course was further complicated by the development of SPHS, septic shock, AKI, and severe thrombocytopenia, necessitating critical care support. Interestingly, she had no signs of jaundice or severe hepatic dysfunction. Initial broad-spectrum therapy with piperacillin-tazobactam failed to achieve clinical improvement. Given her worsening clinical condition, antimicrobial therapy was escalated to meropenem alongside methylprednisolone, which led to the progressive resolution of her pulmonary and renal dysfunction within 48 hours. This report highlights the potential efficacy of meropenem as an alternative antimicrobial therapy for leptospirosis when first-line antibiotics are contraindicated, and supports the role of corticosteroids in leptospirosis-associated pulmonary complications. It also underscores the significance of maintaining a high index of clinical suspicion in high-risk populations to facilitate early diagnosis and antibiotic initiation to improve outcomes in atypical presentations.

## Introduction

Leptospirosis is an infectious zoonosis that has gained significant public health attention owing to its growing morbidity and mortality burden on resource-limited tropical and subtropical regions. The causative agent *Leptospira interrogans*, known to cause clinical disease in humans, is harbored by a range of domestic and wild animals, including rodents, cats, pigs, horses, and cattle. Transmission of leptospirosis to humans occurs through either direct or indirect contact with the urine of infected animals [1,2]. The species *Leptospira interrogans* comprises over 200 serovars. While life-threatening manifestations due to serovar Icterohaemorrhagiae are well-documented [2], the potential of serovar Australis to present with a similarly complicated clinical course has not been extensively reported.

While leptospirosis is classically described as a biphasic illness, atypical presentations are not uncommon. The initial anicteric phase manifests as an acute febrile illness for four to seven days, accompanied by chills, headache, myalgia, abdominal pain, and a pathognomonic conjunctival suffusion. The second immune phase is complicated by severe icteric leptospirosis, which is characterized by multi-organ dysfunction including jaundice, acute kidney injury (AKI), severe pulmonary hemorrhage syndrome (SPHS), acute respiratory distress syndrome (ARDS), myocarditis, uveitis, and rhabdomyolysis [2,3].

Interestingly, severe leptospirosis presenting in the absence of jaundice, a defining feature of Weil’s disease, increases the risks of misdiagnosis and fatal outcomes. Furthermore, pulmonary hemorrhage, occurring in 20-70% of patients, carries a high mortality rate exceeding 60% [4] and is the strongest predictor of poor prognosis. In such cases, prompt clinical recognition and initiation of empirical antibiotics are crucial to curb mortality rates. Leptospirosis is generally susceptible to antibiotics such as penicillin G, third-generation cephalosporins, doxycycline, and macrolides, yet evidence supporting the efficacy of alternative antibiotics in patients allergic to first-line choices remains scarce.

We report the case of a 23-year-old female who was allergic to multiple antibiotics and drugs, presenting with features of severe leptospirosis despite being anicteric, thereby posing unique diagnostic and management challenges.

## Case presentation

A 23-year-old female from the state of Tamil Nadu in India presented to the emergency department with a four-day history of fever. Her symptoms had progressively worsened with generalized myalgia, cough, headache, fatigue, and approximately 20 episodes of vomiting with one episode of blood-stained vomitus. She reported a documented history of allergic reactions to an extensive list of antibiotics, including cephalosporins, penicillins, tetracyclines, macrolides, sulfonamides, as well as nonsteroidal anti-inflammatory drugs (NSAIDs). Similar allergic reactions were also elicited from her family history. She had no significant pre-existing comorbidities nor any recent travel history, yellowish discoloration of the eyes, or bloody sputum.

On admission, she was conscious, alert, pyrexic (100.2 °F) with a heart rate of 103 beats per minute, blood pressure of 100/60 mmHg, and SpO₂ of 98% on room air. Physical examination revealed mild dehydration but no icteric sclera or conjunctival suffusion. Prominent bilateral basal crepitations were heard on auscultation. Neurological and abdominal examinations were unremarkable.

**Figure 1:**
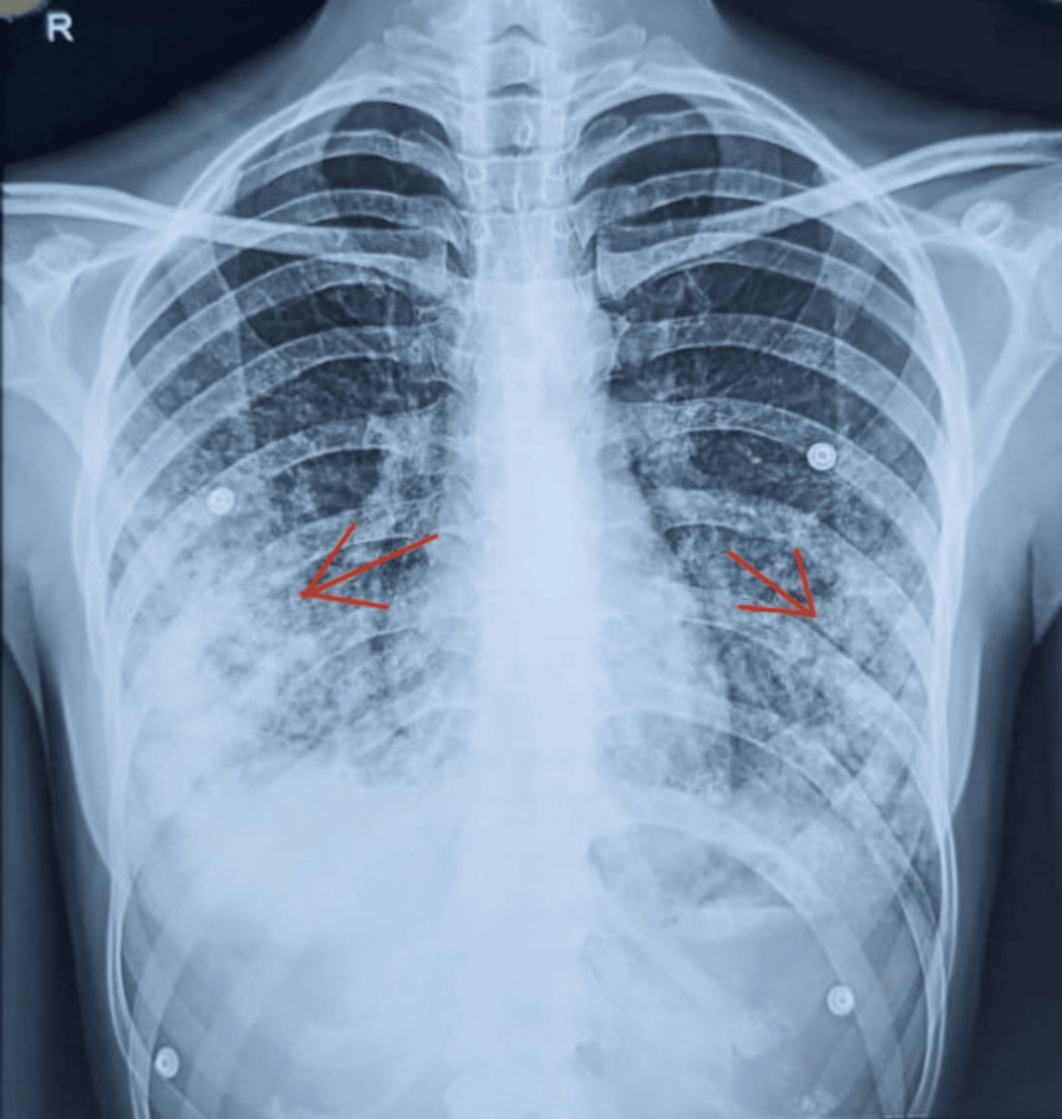
Chest X-ray on admission showing bilateral lower lobe alveolar opacities The image shows alveolar opacities (indicated by red arrows) in the lower lobes of both lungs, indicating a possible pulmonary complication due to leptospirosis and the need for further radiological assessment

Initial laboratory studies (Table 1) revealed anaemia, thrombocytopenia, leukocytosis with neutrophil predominance, AKI, elevated procalcitonin and C-reactive protein (CRP) levels, mild transaminitis, hypoalbuminemia, hyponatremia, and hypokalemia, while urinalysis revealed proteinuria. Extensive workup ruled out differential diagnoses such as dengue, hepatitis, HIV, influenza A and B, and enteric fever. However, the microscopic agglutination test (MAT) revealed a significant titre (1:160) for *Leptospira interrogans* serovar Australis. Chest X-ray showed bilateral alveolar opacities in both lower lobes (Figure 1). Ultrasonography demonstrated increased renal cortical echoes, and the echocardiogram was unremarkable. Test doses were administered for all medications before commencement, and she developed allergic rashes to doxycycline, azithromycin, ceftriaxone, cefotaxime, penicillin G, and co-amoxiclav. She was empirically started on intravenous piperacillin-tazobactam 4.5 g twice daily after tolerating the test dose well, and was closely monitored for any allergic or Jarisch-Herxheimer reaction.

**Table 1:** Laboratory parameters at presentation WBC: white blood cell count; CRP: C-reactive protein;

Parameters	Result	Reference range
Hemoglobin, g/dL	10.1	11.5-16.0
Platelet count, cells/mm³	46,000	150,000-400,000
WBC, cells/mm³	18,050	4000-11,000
Neutrophil %	91	45-70
Blood urea, mg/dL	67	10-45
Creatinine, mg/dL	2.9	0.6-1.2
Procalcitonin, ng/mL	1.13	<0.5
CRP, mg/L	36.7	<5
Sodium, mmol/L	131	130-145
Potassium, mmol/L	2.9	3.5-5.0
Aspartate transaminase, U/L	135	5-45
Alanine transaminase, U/L	56	5-33
Serum albumin, g/dL	3.2	3.8-5.4

**Figure 2:**
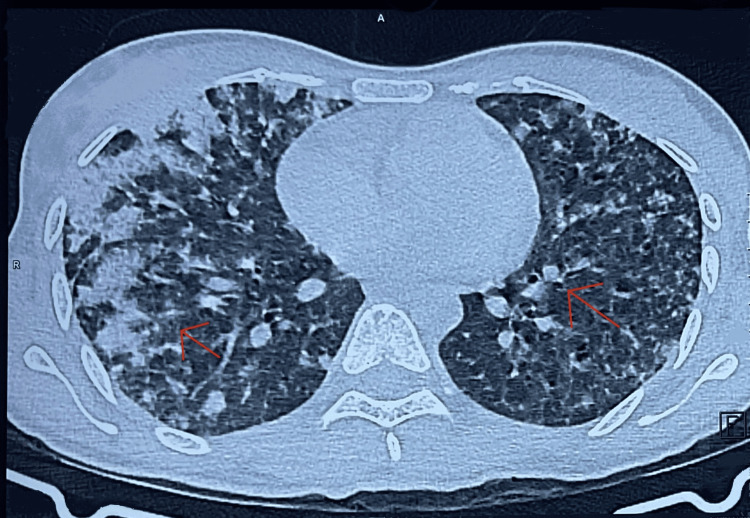
HRCT scan of the thorax showing bilateral dense alveolar opacities suggestive of SPHS The image shows multiple dense alveolar opacities (indicated by red arrows) predominantly in the lower lobes of both lungs, suggestive of alveolar hemorrhage secondary to severe leptospirosis HRCT: high-resolution computed tomography; SPHS: severe pulmonary hemorrhage syndrome

On day two of admission, the patient developed hemoptysis, septic shock, and ARDS. She was transferred to the ICU, where she required intermittent bi-level positive airway pressure (BiPAP) support, high-flow oxygen, fluid challenge, and intravenous tranexamic acid. High-resolution CT (HRCT) of the thorax (Figure 2) revealed bilateral dense alveolar opacities suggestive of SPHS. In view of pulmonary hemorrhage and worsening renal function, serological studies were done for anti-neutrophil cytoplasmic antibody (ANCA)-associated vasculitis, systemic lupus erythematosus, and Goodpasture syndrome, all of which were negative. Blood and urine cultures yielded no growth. A working diagnosis of leptospirosis-induced multi-organ dysfunction syndrome (MODS) was established.

**Figure 3:**
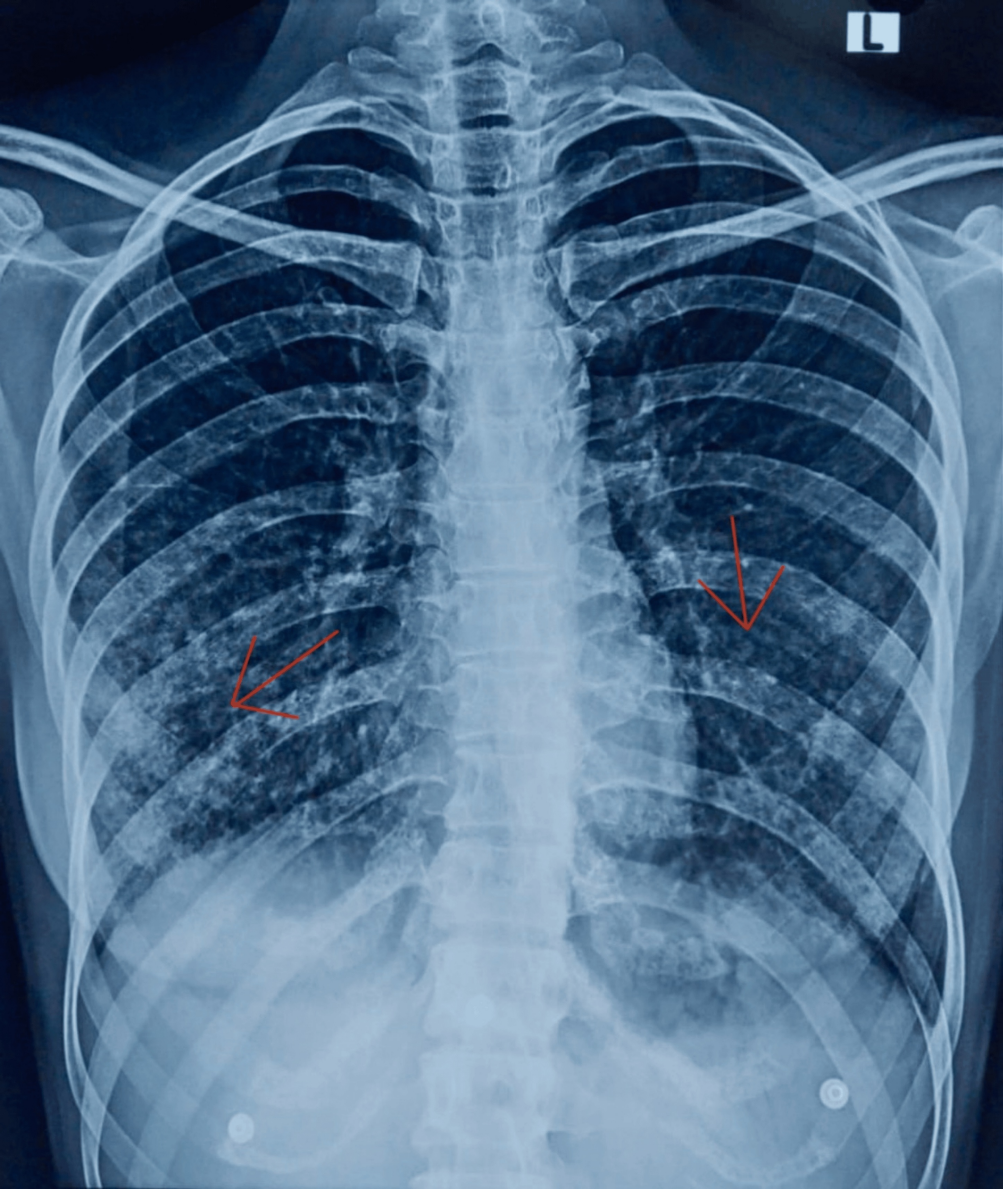
Repeat chest X-ray showing clearance of alveolar opacities The image shows resolution of alveolar opacities (indicated by red arrows) in the lower lobes of both lungs, correlating with clinical improvement after treatment with meropenem and corticosteroids

Antimicrobial treatment was stepped up to intravenous meropenem (1 g stat dose followed by 500 mg every eight hours) with intravenous methylprednisolone 80 mg twice daily. Due to severe thrombocytopenia on day two of admission (Table 2), she received one unit of single donor platelet transfusion. Although her platelet counts improved, she developed pedal edema with an elevated serum creatinine level of 3.7 mg/dL, and she was counseled on the potential need for hemodialysis. Fortunately, her creatinine level improved on day three of admission. On day four of admission, her clinical condition substantially improved, and her repeat chest X-ray (Figure 3) showed clearance of alveolar opacities. She was shifted to the medical ward and continued on meropenem for seven days with tapering doses of methylprednisolone. Manual platelet count and other laboratory tests done on day seven of admission were within normal limits, and she was symptomatically well. She was discharged on oral faropenem 200 mg and deflazacort 8 mg twice daily for five days. Upon follow-up five days after discharge, she continued to recover well with a normal complete blood count. 

**Table 2:** All investigations during hospital stay WBC: white blood cell count; CRP: C-reactive protein

Parameters	Day 1 (admission)	Day 2	Day 3	Day 4	Day 5	Day 7	Reference range
Platelet count, cells/mm³	46,000	17,000	51,000	50,000	50,000	N/A	150,000-400,000
WBC, cells/mm³	18,050	19,170	14,100	13,230	12,600	N/A	4000-11,000
Blood urea, mg/dL	67	96	104	80	52	21	10-45
Creatinine, mg/dL	2.9	3.7	3.1	1.5	1	0.6	0.6-1.2
Sodium, mmol/L	131	133	130	132	133	133	130-145
Potassium, mmol/L	2.9	3.2	3.3	2.9	3.2	3.7	3.5-5.0
CRP, mg/L	36.7	N/A	N/A	N/A	6.6	N/A	<5
Manual platelet count, cells/mm³	N/A	N/A	N/A	N/A	87,000	171,000	150,000-400,000
Aspartate transaminase, U/L	135	N/A	24	N/A	N/A	28	5-45
Alanine transaminase, U/L	56	N/A	40	N/A	N/A	34	5-33
Serum albumin, g/dL	3.2	N/A	3	N/A	N/A	3.1	3.8-5.4

## Discussion

Leptospirosis is one of the most prevalent zoonotic diseases globally, but is often underdiagnosed due to its nonspecific clinical manifestations and variable spectrum of severity. The pathogenic spirochete in humans, *Leptospira interrogans*, has been classified into more than 200 serovars within over 20 serogroups based on agglutination after cross-absorption with a homologous antigen [2]. The most common *Leptospira* serovars responsible for human infection are Icterohaemorrhagiae, Australis, Autumnalis, Grippotyphosa, Canicola, and Pomona. Serovar Icterohaemorrhagiae has been classically linked with the development of lethal clinical complications seen in leptospirosis, as described subsequently, and associated with the severe variant of leptospirosis - clinically known as Weil’s disease [5]. Interestingly, in our case, the identified pathogenic organism was *Leptospira interrogans *serovar Australis, which is not classically documented as a lethal serovar. Thus, our case contributes to the epidemiological body of knowledge on other serovars, such as Australis, and further highlights the potential for serovars other than Icterohaemorrhagiae to present with fatal complications of the disease.

A systematic review estimated that over 73% of cases and deaths in leptospirosis occur between the Tropics of Cancer and Capricorn, highlighting the burden of leptospirosis on underdeveloped and developing tropical countries [6]. Leptospirosis is notably endemic in the Indian subcontinent, with cases being predominantly reported from southern states like Tamil Nadu [7] owing to its tropical climate, frequent floods, inadequate sanitary standards, widespread farming and animal husbandry practices. Leptospires are maintained in the renal tubules of infected animals and shed in their urine. Humans are accidental hosts who can acquire the infection from direct handling of animals via skin breaches or mucous membranes, or by indirect contact with contaminated water, food, and soil. Occupations such as agricultural and livestock farmers, sewage workers, meat and animal handlers, and veterinarians are the most significant groups at risk [2]. Recreational exposure includes water sports and practices of swimming and bathing in rural India’s water bodies. Rare modes of transmission, such as animal bites and sexual transmission between humans, have been reported [8]. In our case, the patient denied any significant occupational or recreational exposure to a potential source of infection, but presented with life-threatening features. This emphasises the significance of considering leptospirosis in a differential diagnosis in patients from highly endemic areas, regardless of their exposure history.

The course of clinical presentation in leptospirosis is typically described as biphasic. The initial anicteric phase of infection, characterised by the presence of leptospires in blood, presents as an acute febrile illness and non-specific constitutional, respiratory, and gastrointestinal symptoms, which include but are not limited to chills, myalgia, headache, conjunctival suffusion, abdominal pain, and rarely a transient rash [1]. This is followed by an immune phase in the second week of illness, which is associated with antibody production and isolation of leptospires in urine. Conjunctival suffusion occurring simultaneously with scleral icterus is considered pathognomonic for Weil’s disease [2]. In our case, the patient initially presented with a history of acute febrile illness, with associated complaints of cough, myalgia, headache, and multiple vomiting episodes, including one episode of blood-stained vomitus. However, signs of potential severity, such as conjunctival suffusion and scleral icterus, were not present on initial assessment in this patient. Subsequently, during the second phase, a complicated clinical course ensues in about 10% of patients due to underlying immune-mediated mechanisms.

Severe leptospirosis is traditionally synonymous with Weil’s disease, and characterised by jaundice and AKI, potentially alongside other systemic complications such as SPHS, thrombocytopenia, myocarditis, gastrointestinal bleeding, pancreatitis, and aseptic meningitis. Leptospirosis-induced AKI is typically a potassium-wasting non-oliguric renal failure secondary to direct nephrotoxicity of leptospires on proximal tubules and immune-mediated inflammatory damage, necessitating intravenous fluid and potassium replacement [9]. SPHS, manifesting radiologically as diffuse alveolar hemorrhage, has emerged as a leading cause of death with mortality rates exceeding 50%. The severity of pulmonary disease in leptospirosis is notably unrelated to the presence of jaundice, whereas cigarette smoking is considered a significant risk factor for its development. SPHS is thought to be caused by bacterial disruption of the endothelial integrity of alveolar capillaries and a host-mediated pro-inflammatory cytokine storm [10]. Clinically, patients present with a range of pulmonary manifestations that include cough, dyspnea, hemoptysis, and can progress to acute respiratory distress syndrome.

In addition to renal and pulmonary complications, previous literature has demonstrated jaundice to be prevalent in more than 60% of severe leptospirosis cases [11] and shown that hyperbilirubinemia and jaundice are considered to be the chief mortality predictors [12]. However, anicteric cases can occur in the presence of other severe life-threatening complications associated with leptospirosis and could lead to a misdiagnosis in many cases, and thus increase mortality risks [1]. In our case, although there was no evidence of severe liver dysfunction or clinical jaundice, the patient developed clinical and radiological evidence of SPHS, which included worsening pulmonary symptoms, hemoptysis, acute respiratory distress, and diffuse alveolar hemorrhage, coupled with other complications associated with severe leptospirosis such as AKI, thrombocytopenia, and septic shock. Of particular note in our case, despite the absence of jaundice or hyperbilirubinemia, the complications emerging in the patient’s course of illness, especially SPHS, AKI, septic shock, and thrombocytopenia, were all independent predictors of death [13]. This underscores the need for a high index of suspicion for severe leptospirosis even when jaundice is not clinically evident, especially in high-risk endemic regions, to aid early laboratory confirmation and prompt initiation of treatment that could prove to be life-saving.

Early diagnosis for empirical initiation of effective antimicrobial and supportive treatment is the strongest predictor for survival in severe cases. Leukocytosis, thrombocytopenia, and raised inflammatory markers are particular laboratory indicators that help distinguish leptospirosis from other viral causes of acute febrile illness [2]. Our patient exhibited all three findings, raising our clinical suspicion of leptospirosis. We used the MAT, a gold standard diagnostic investigation that detects serovar-specific antibodies, and confirmed the presence of *L. interrogans* serovar Australis. However, serological diagnosis can be challenging in the first week of illness due to a lack of antibody production. Research recommends that in the early phase, a combined strategy utilising molecular PCR testing and IgM-Enzyme-linked immunosorbent assay (ELISA) or MAT is superior [14]. Alternatively, we also recommend the modified Faine’s criteria in highly endemic areas to establish a diagnosis based on clinical, epidemiological, and laboratory data. In this case, these criteria were vital in formulating a presumptive diagnosis of leptospirosis on the basis of the patient receiving a score of 23.

Upon suspecting leptospirosis, empirical treatment should commence with any of the recommended first-line antibiotics, which include penicillin G, doxycycline, ampicillin, ceftriaxone, cefotaxime, oxytetracycline, and azithromycin [1]. Other supportive management crucial to survival includes tackling complications by timely hemodialysis for AKI, ventilatory support in ARDS, and plasmapheresis in refractory cases. We encountered a significant challenge in managing our patient owing to the extensive history of documented drug allergies, which prevented our use of any of these first-line choices. Piperacillin-tazobactam, although initially trialled in this case, showed a lack of clinical improvement and rising leukocyte counts, warranting a shift to meropenem, which was found to significantly improve the patient’s condition and labs within 48 hours.

Although monotherapy with meropenem was effective in treating our case, it is not a standard first-line antimicrobial agent for severe leptospirosis. Meropenem has been reported to have been used in combination with other antibiotics, particularly in settings of severe pulmonary manifestations of leptospirosis and co-infection with scrub typhus. However, the use of meropenem as monotherapy for leptospirosis has yet to show efficacy in existing literature, and further research will be required to validate its use, especially in settings where first-line choices are unavailable or contraindicated, as seen in our case. The rationale behind the use of corticosteroids in treating severe pulmonary manifestations of leptospirosis is to suppress the immune-mediated inflammatory cascade that contributes to multi-organ dysfunction. The literature has contradictory information on the use of steroids in patients with pulmonary leptospirosis.

Some studies have observed improved outcomes with the use of steroids in patients with pulmonary complications. In one particular case series [15], a regimen of 1g bolus methylprednisolone for three days followed by oral prednisolone 1mg/kg/day for seven days was used and showed a reduction in mortality rates when started early in the course of management. However, another study found no significant benefit and also the development of nosocomial infections in patients receiving dexamethasone for pulmonary leptospirosis, highlighting the potential for negative patient outcomes and increased mortality risks with steroid initiation [16]. However, methylprednisolone showed remarkable results in our case, resolving the patient’s pulmonary hemorrhage and septic shock within 48 hours. This highlights the need for further research to clearly demarcate the benefits and risks of using steroids in the treatment of severe cases of leptospirosis.

## Conclusions

Leptospirosis, although typically an acute undifferentiated febrile illness, can have varying manifestations. Our patient presented with complications secondary to leptospirosis, but lacked the characteristic jaundice seen typically in these patients, creating diagnostic uncertainty. Thus, in endemic regions, heightened clinical suspicion when encountering atypical presentations is crucial for early diagnosis and antibiotic initiation, both of which can improve patient outcomes. In this patient, meropenem proved to be effective in resolving the pulmonary and renal complications caused by severe leptospirosis. Nevertheless, the challenges posed by the patient’s allergic history to first-line antibiotics highlight the need for further research to establish the efficacy of non-standard antimicrobial alternatives such as meropenem and piperacillin-tazobactam. Furthermore, guidelines resolving the controversy around the use of corticosteroids in patients with severe leptospirosis need to be established, while future studies also need to explore the clinical spectrum of various serovars to build further on our epidemiological understanding of leptospirosis.
